# Complete genomes of 568 diverse *Klebsiella pneumoniae* species complex isolates from humans, animals, and marine sources in Norway from 2001 to 2020

**DOI:** 10.1128/mra.00931-24

**Published:** 2025-05-22

**Authors:** Marit A. K. Hetland, Mia A. Winkler, Håkon Kaspersen, Fredrik Håkonsholm, Ragna-Johanne Bakksjø, Eva Bernhoff, Jose F. Delgado-Blas, Sylvain Brisse, Annapaula Correia, Aasmund Fostervold, Margaret M. C. Lam, Bjørn-Tore Lunestad, Nachiket P. Marathe, Niclas Raffelsberger, Ørjan Samuelsen, Marianne Sunde, Arnfinn Sundsfjord, Anne Margrete Urdahl, Ryan R. Wick, Kathryn E. Holt, Iren H. Löhr

**Affiliations:** 1 Department of Medical Microbiology, Stavanger University Hospital 60496, Stavanger, Norway; 2 Department of Biological Sciences, Faculty of Science and Technology, University of Bergen 123257, Bergen, Norway; 3 Department of Medical Biology, Faculty of Health Sciences, UiT - The Arctic University of Norway 60482, Tromsø, Norway; 4 Research Section Food Safety and Animal Health, Department of Animal Health and Food Safety, Norwegian Veterinary Institute 87573, Ås, Norway; 5 Institute of Marine Research 115347 , Bergen, Norway; 6 Biodiversity and Epidemiology of Bacterial Pathogens Unit, Institut Pasteur, Université Paris Cité 555089, Paris, France; 7 Department of Infection Biology, Faculty of Infectious and Tropical Diseases, London School of Hygiene & Tropical Medicine 270390, London, United Kingdom; 8 Department of Clinical Science, Faculty of Medicine, University of Bergen 542304, Bergen, Norway; 9 Department of Infectious Diseases, School of Translational Medicine, Monash University 589641 , Melbourne, Victoria, Australia; 10 Department of Microbiology and Infection Control, University Hospital of North Norway 60519, Tromsø, Norway; 11 Norwegian National Advisory Unit on Detection of Antimicrobial Resistance, Department of Microbiology and Infection Control, University Hospital of North Norway 60519, Tromsø, Norway; 12 Section for Bacteriology, Department for Analysis and Diagnostics, Norwegian Veterinary Institute 87573, Ås, Norway; 13 Department of Microbiology and Immunology, University of Melbourne at the Peter Doherty Institute for Infection and Immunity 534133 , Melbourne, Victoria, Australia; The University of Arizona, Tucson, Arizona, USA

**Keywords:** *Klebsiella pneumoniae*, hybrid assembly, ONT, Illumina, One Health, complete genomes

## Abstract

We report 578 hybrid genome assemblies (568 complete) of *Klebsiella pneumoniae* species complex isolates from human, animal, and marine sources in Norway collected from 2001 to 2020, belonging to five phylogroups including *K. pneumoniae* (n = 492) and *K. variicola* (n = 69) and 364 unique sequence types.

## ANNOUNCEMENT

**Fig 1:**
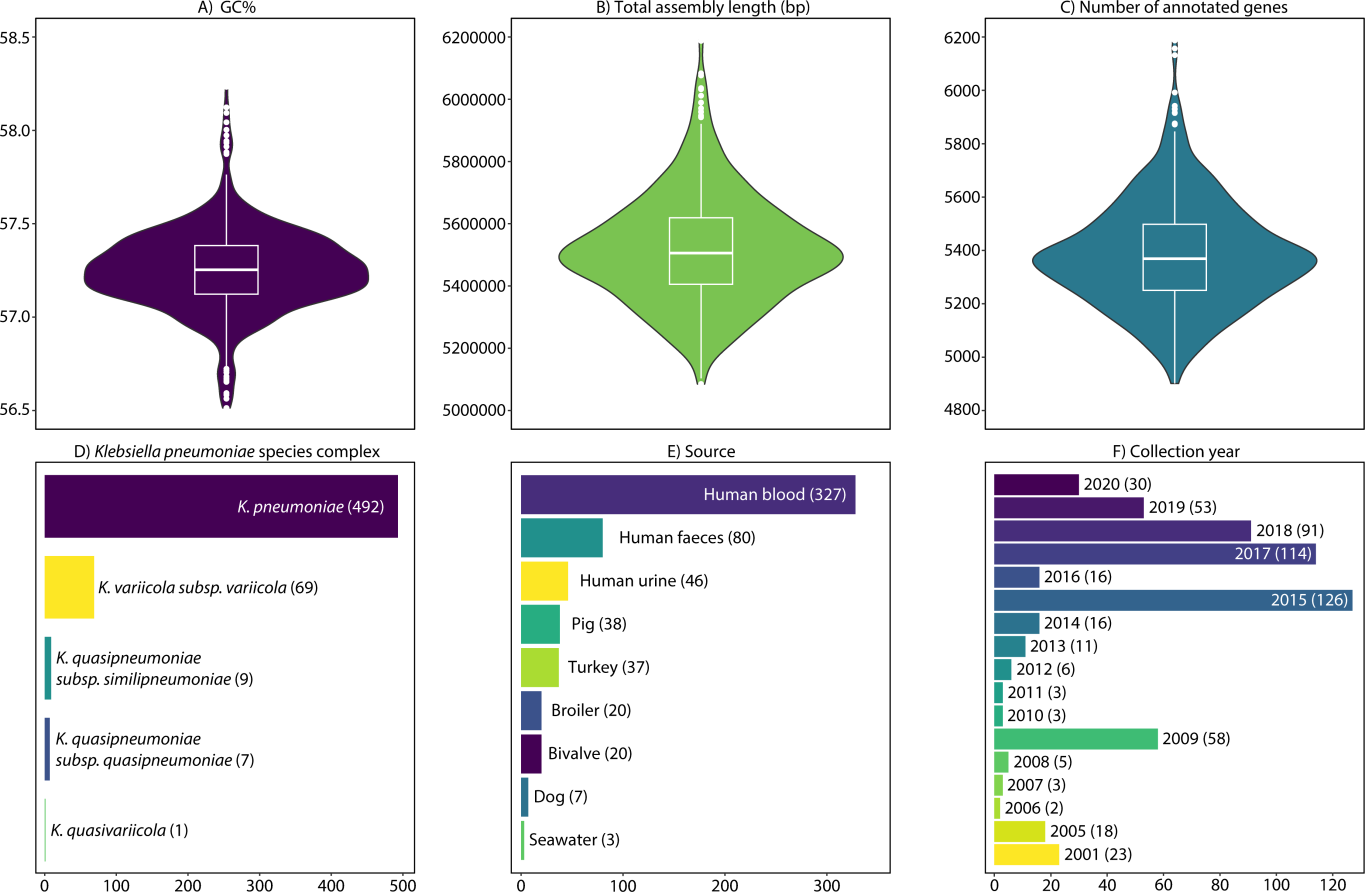
Quality metrics and description of the 578 (568 complete) hybrid-assembled *Klebsiella pneumoniae* species complex (KpSC) genomes, showing the distributions of (**A**) GC%, (**B**) total assembly length, (**C**) number of annotated genes, (**D**) KpSC phylogroup, (**E**) source type, and (**F**) year of collection.

*Klebsiella pneumoniae* species complex are opportunistic pathogens that can transmit between humans, animals, and the environment (1). Here, we report hybrid genome assemblies of 578 (568 complete) genetically diverse isolates from human, animal, and marine sources (Fig. 1).

The isolates were obtained as described previously (2–10) (see also Table 1; the full version is available on Figshare at https://doi.org/10.6084/m9.figshare.c.7622345). They were received in frozen stock, then plated on blood agar, incubated overnight at 35°C, and DNA was extracted from a representative sweep of multiple colonies using a 10 µL Sarstedt inoculation loop. Approximately one-fourth of the loop volume was used. For short-read sequencing, DNA was isolated and purified using the MagNA Pure 96 DNA and Viral NA Small Volume Kit on the MagNA Pure 96 System (Roche Diagnostics, Rotkreutz, Switzerland). Libraries were prepared with Illumina DNA prep or Nextera XT DNA Library preparation kits and protocols (Illumina Inc, San Diego, USA), as specified in Table 1. Sequencing was performed on the Illumina MiSeq (Illumina Inc, San Diego, USA), using MiSeq Reagents kit v2 or v3 sequencing chemistry, generating 2 × 150 bp, 2 × 250 bp, or 2 × 300 bp paired-end reads. Twelve isolates were sequenced on the Illumina HiSeq 2500, generating 2 × 125 bp paired-end reads. Different genomic DNA extractions were used for the Illumina and ONT read libraries, except for a few isolates where replicons were only resolved after sequencing with the same extraction. The FASTQ files were quality-filtered with TrimGalore v0.6.7 (https://github.com/FelixKrueger/TrimGalore). For long-read sequencing, DNA was extracted using the GenFind V3 reagent kit with the Bacteria protocol (Beckman Coulter Life Sciences, Indianapolis, USA). Library preparation was performed with the ligation (SQK-LSK-109) or rapid (SQK-RBK114-24) kits (Oxford Nanopore Technologies [ONT], Oxford, UK). DNA libraries were then loaded onto R9.4.1 (*n* = 533) or R10.4.1 (*n* = 45) MinION flow cells and sequenced on the ONT GridION, aiming for ≥ 100X average read depth. The fast5 files were basecalled with Guppy v6.4.2+97a7f06, using the super-accurate basecalling model. The FASTQ files were subsampled with Filtlong v0.2.1 (https://github.com/rrwick/Filtlong), discarding reads shorter than 1 kbp and removing the 5% lowest-quality reads.

We performed both long-read-first (Flye v2.9-b1768 (11)) and short-read-first (Unicycler v0.5.0 (12)) hybrid assembly. Flye does not assemble small plasmids (<10 Kbp) as well as Unicycler (13). However, Unicycler employs a contamination filter, which can exclude low-copy-number plasmids. We therefore used both approaches to maximize plasmid recovery. Using clinopore-nf v1.1 (https://github.com/HughCottingham/clinopore-nf), the genomes were assembled with Flye and polished with Medaka v1.5.0 (https://github.com/nanoporetech/medaka), Polypolish v0.5.0 (14), and POLCA v4.0.5 (15). The same genomes were assembled with Unicycler, using SPAdes v3.15.4 (16), and polished using the same tools. Trycycler v0.5.3 (17) was used to cluster plasmid contigs (<1 Mbp) from the two assemblers by a mash distance of ≤ 0.01. If a plasmid was found by both assemblers, both were closed and of similar length, we kept the one from Flye; if a plasmid was found by only one assembler, we kept it. All plasmids were rotated with Circlator v1.5.5 (18).

Default parameters were used for all methods, except where otherwise noted. Assembly quality was assessed with fast_count (https://github.com/rrwick/MinION-desktop), FastQC v0.11.9 (https://www.bioinformatics.babraham.ac.uk/projects/fastqc/), Quast v5.2.0 (19), and Kleborate v2.4.1 (20) (Fig. 1 and Table 1). The replicons of 568 genomes were fully closed (circularized or linear), and the remaining 10 had unclosed plasmids. Overall, 578 chromosomes (all closed) and 1,428 plasmid contigs were assembled (1,416 circularized, one linear, and 11 not closed), with a mean of 2.45 plasmids per genome (range 0–10). The genomes were annotated using NCBI’s prokaryotic assembly pipeline PGAP v6.5–6.8 (21). The pangenome was inferred with Panaroo v1.3.3 (22), revealing 31,417 unique genes, of which 3,036 were core, i.e., shared by all 578 isolates.

**TABLE 1:** Genome quality and metadata (abridged; full version available on Figshare at 10.6084 /m9.figshare.c.7622345)

Strain	Phylogroup ^*a*^	SL	Collection year	Host	Source	BioSample accession	Strain (continued)	Phylogroup (continued)	SL (continued)	Collection year (continued)	Host (continued)	Source (continued)	BioSample accession (continued)
2018–01-2353	Kp1	SL11172	2018	Broiler	Cecal	SAMEA8399304	NK_H22_018	Kp3	SL10012	2017	Human	Blood	SAMEA114492477
2018–01-2734	Kp1	SL11217	2018	Broiler	Cecal	SAMEA8399335	NK_H22_027	Kp1	SL17	2017	Human	Blood	SAMEA113605861
2018–01-2782	Kp1	SL290	2018	Broiler	Cecal	SAMEA8399337	NK_H22_045	Kp1	SL380	2017	Human	Blood	SAMEA113605878
2018–01-3723	Kp1	SL661	2018	Broiler	Cecal	SAMEA8399398	NK_H22_053	Kp1	SL251	2017	Human	Blood	SAMEA113605884
2018–01-389	Kp1	SL35	2018	Broiler	Cecal	SAMEA8399414	NK_H22_060	Kp1	SL244	2017	Human	Blood	SAMEA113605890
2018–01-4203	Kp1	SL10066	2018	Broiler	Cecal	SAMEA8399433	NK_H22_070	Kp1	SL11308	2018	Human	Blood	SAMEA113605899
2018–01-4639	Kp1	SL463	2018	Broiler	Cecal	SAMEA8399445	NK_H22_075	Kp1	SL462	2018	Human	Blood	SAMEA113605904
2018–01-710	Kp1	SL10868	2018	Broiler	Cecal	SAMEA8399471	NK_H22_076	Kp1	SL37	2018	Human	Blood	SAMEA113605905
2018–01-711	Kp1	SL11049	2018	Broiler	Cecal	SAMEA8399472	NK_H23_002	Kp1	SL107	2017	Human	Blood	SAMEA113605925
2018–01-714	Kp1	SL17	2018	Broiler	Cecal	SAMEA8399473	NK_H23_005	Kp1	SL3598	2017	Human	Blood	SAMEA113605928
KGVET-2020–01-2318-1	Kp1	SL3020	2020	Broiler	Cecal	SAMN32676066	NK_H23_006	Kp1	SL45	2017	Human	Blood	SAMEA113605929
KGVET-2020–01-2328-1	Kp3	SL10792	2020	Broiler	Cecal	SAMN32676067	NK_H23_007	Kp1	SL14	2017	Human	Blood	SAMEA113605930
KGVET-2020–01-2846-1	Kp1	SL10139	2020	Broiler	Cecal	SAMN32676088	NK_H23_014	Kp1	SL882	2017	Human	Blood	SAMEA113605937
KGVET-2020–01-3513-1	Kp1	SL37	2020	Broiler	Cecal	SAMN32676116	NK_H23_015	Kp1	SL23	2017	Human	Blood	SAMEA113605938
KGVET-2020–01-3514	Kp1	SL2513	2020	Broiler	Cecal	SAMN32676117	NK_H23_019	Kp1	SL133	2017	Human	Blood	SAMEA113605942
KGVET-2020–01-3515	Kp1	SL10870	2020	Broiler	Cecal	SAMN32676118	NK_H23_027	Kp1	SL107	2018	Human	Blood	SAMEA113605949
KGVET-2020–01-4188-1	Kp1	SL292	2020	Broiler	Cecal	SAMN32676136	NK_H23_041	Kp1	SL268	2018	Human	Blood	SAMEA113605963
KGVET-2020–01-4327	Kp1	SL10334	2020	Broiler	Cecal	SAMN32676144	NK_H23_045	Kp1	SL234	2018	Human	Blood	SAMEA113605967
KGVET-2020–01-468-1	Kp1	SL37	2020	Broiler	Cecal	SAMN32676150	NK_H23_049	Kp1	SL29	2018	Human	Blood	SAMEA113605970
KGVET-2018–04-44577	Kp1	SL200	2018	Broiler	Spleen	SAMEA115162996	NK_H24_006	Kp1	SL11126	2017	Human	Blood	SAMEA113605977
KGMAR-2016–319	Kp1	SL10077	2016	*C. gigas*		SAMN13382562	NK_H24_010	Kp1	SL107	2017	Human	Blood	SAMEA113605981
KGMAR-2020–314	Kp1	SL3010	2020	*C. gigas*		SAMN17766502	NK_H24_016	Kp1	SL2701	2017	Human	Blood	SAMEA113605987
KGVET-2020–01-1661	Kp1	SL1583	2020	Dog	Ear	SAMEA115162999	NK_H24_028	Kp1	SL307	2017	Human	Blood	SAMEA113605997
KGVET-2019–01-2264	Kp1	SL186	2019	Dog	Fecal	SAMEA114492489	NK_H24_044	Kp1	SL200	2018	Human	Blood	SAMEA113606012
KGVET-2019–01-3472-4	Kp1	SL10871	2019	Dog	Fecal	SAMEA115162997	NK_H24_054	Kp1	SL405	2018	Human	Blood	SAMEA113606021
KGVET-2019–01-3619	Kp1	SL34	2019	Dog	Fecal	SAMEA115374738	NK_H25_002	Kp1	SL268	2017	Human	Blood	SAMEA113606025
KGVET-2019–01-792	Kp3	SL10629	2019	Dog	Fecal	SAMEA115162998	NK_H25_013	Kp1	SL133	2018	Human	Blood	SAMEA113606035
KGVET-2018–01-3794	Kp1	SL10176	2018	Dog	Lung	SAMEA115162995	NK_H26_006	Kp1	SL188	2017	Human	Blood	SAMEA113605196
KGVETS-2019–01-4848	Kp1	SL37	2019	Dog	Skin	SAMEA115163001	NK_H28_005	Kp1	SL107	2018	Human	Blood	SAMEA113605458
KP_NORM_BLD_1032	Kp1	SL420	2005	Human	Blood	SAMEA8948184	NK_H29_003	Kp3	SL11167	2017	Human	Blood	SAMEA113605471
KP_NORM_BLD_110772	Kp1	SL846	2015	Human	Blood	SAMEA8948730	NK_H3_001	Kp1	SL17	2017	Human	Blood	SAMEA113605100
KP_NORM_BLD_111588	Kp1	SL25	2015	Human	Blood	SAMEA8948740	NK_H3_002	Kp1	SL294	2017	Human	Blood	SAMEA113605101
KP_NORM_BLD_111598	Kp3	SL925	2015	Human	Blood	SAMEA8948743	NK_H3_007	Kp1	SL17	2018	Human	Blood	SAMEA111533203
KP_NORM_BLD_112590	Kp1	SL34	2015	Human	Blood	SAMEA8948756	NK_H3_009	Kp1	SL3659	2017	Human	Blood	SAMEA113605106
KP_NORM_BLD_112596	Kp1	SL1626	2015	Human	Blood	SAMEA8948757	NK_H3_015	Kp1	SL628	2018	Human	Blood	SAMEA113605112
KP_NORM_BLD_112603	Kp1	SL10	2015	Human	Blood	SAMEA8948759	NK_H3_019	Kp1	SL3010	2018	Human	Blood	SAMEA113605116
KP_NORM_BLD_112904	Kp1	SL3010	2015	Human	Blood	SAMEA8948761	NK_H3_025	Kp1	SL107	2018	Human	Blood	SAMEA113605121
KP_NORM_BLD_112908	Kp1	SL1630	2015	Human	Blood	SAMEA8948762	NK_H4_002	Kp1	SL54	2017	Human	Blood	SAMEA113605123
KP_NORM_BLD_112911	Kp3	SL3937	2015	Human	Blood	SAMEA8948763	NK_H4_006	Kp3	SL10047	2017	Human	Blood	SAMEA113605127
KP_NORM_BLD_112916	Kp1	SL33	2015	Human	Blood	SAMEA8948765	NK_H4_026	Kp1	SL37	2018	Human	Blood	SAMEA113605144
KP_NORM_BLD_112922	Kp1	SL34	2015	Human	Blood	SAMEA8948767	NK_H5_007	Kp1	SL502	2017	Human	Blood	SAMEA113605157
KP_NORM_BLD_112925	Kp1	SL180	2015	Human	Blood	SAMEA8948768	NK_H5_010	Kp1	SL107	2017	Human	Blood	SAMEA113605160
KP_NORM_BLD_113315	Kp1	SL23	2015	Human	Blood	SAMEA8948777	NK_H5_014	Kp1	SL3010	2017	Human	Blood	SAMEA113605164
KP_NORM_BLD_113321	Kp1	SL17	2015	Human	Blood	SAMEA8948778	NK_H5_016	Kp1	SL186	2017	Human	Blood	SAMEA113605166
KP_NORM_BLD_113768	Kp3	SL363	2015	Human	Blood	SAMEA8948784	NK_H5_031	Kp1	SL258	2018	Human	Blood	SAMEA113605180
KP_NORM_BLD_113888	Kp1	SL37	2015	Human	Blood	SAMEA8948794	NK_H7_005	Kp1	SL3010	2017	Human	Blood	SAMEA113605213
KP_NORM_BLD_114309	Kp3	SL596	2015	Human	Blood	SAMEA8948813	NK_H7_010	Kp1	SL147	2018	Human	Blood	SAMEA113605218
KP_NORM_BLD_114453	Kp3	SL3235	2015	Human	Blood	SAMEA8948829	NK_H8_002	Kp1	SL107	2017	Human	Blood	SAMEA113605223
KP_NORM_BLD_114458	Kp1	SL15	2015	Human	Blood	SAMEA8948830	NK_H8_008	Kp1	SL461	2017	Human	Blood	SAMEA113605229
KP_NORM_BLD_114473	Kp2	SL526	2015	Human	Blood	SAMEA8948834	NK_H8_011	Kp1	SL321	2017	Human	Blood	SAMEA113605232
KP_NORM_BLD_114482	Kp3	SL11198	2015	Human	Blood	SAMEA8948837	NK_H8_022	Kp1	SL107	2017	Human	Blood	SAMEA113605243
KP_NORM_BLD_114487	Kp1	SL101	2015	Human	Blood	SAMEA8948839	NK_H8_044	Kp1	SL35	2017	Human	Blood	SAMEA113605263
KP_NORM_BLD_114603	Kp1	SL34	2015	Human	Blood	SAMEA8948842	NK_H8_045	Kp3	SL919	2017	Human	Blood	SAMEA113605264
KP_NORM_BLD_115332	Kp1	SL37	2015	Human	Blood	SAMEA8948851	NK_H8_052	Kp1	SL10554	2017	Human	Blood	SAMEA113605271
KP_NORM_BLD_115351	Kp1	SL17	2015	Human	Blood	SAMEA8948855	NK_H8_057	Kp1	SL107	2017	Human	Blood	SAMEA113605275
KP_NORM_BLD_115369	Kp1	SL37	2015	Human	Blood	SAMEA8948859	NK_H8_060	Kp1	SL111	2017	Human	Blood	SAMEA113605278
KP_NORM_BLD_115706	Kp3	SL641	2015	Human	Blood	SAMEA8948874	NK_H8_070	Kp1	SL10873	2018	Human	Blood	SAMEA113605287
KP_NORM_BLD_115796	Kp1	SL11174	2015	Human	Blood	SAMEA8948884	NK_H8_077	Kp1	SL10	2018	Human	Blood	SAMEA113605294
KP_NORM_BLD_116239	Kp1	SL37	2015	Human	Blood	SAMEA8948902	NK_H8_083	Kp1	SL45	2018	Human	Blood	SAMEA113605300
KP_NORM_BLD_116241	Kp1	SL11502	2015	Human	Blood	SAMEA8948903	NK_H8_100	Kp1	SL3010	2018	Human	Blood	SAMEA113605314
KP_NORM_BLD_116378	Kp1	SL11152	2015	Human	Blood	SAMEA8948920	NK_H8_111	Kp1	SL107	2018	Human	Blood	SAMEA113605325
KP_NORM_BLD_116392	Kp1	SL147	2015	Human	Blood	SAMEA8948923	NK_H8_114	Kp1	SL37	2018	Human	Blood	SAMEA113605328
KP_NORM_BLD_116566	Kp1	SL10833	2015	Human	Blood	SAMEA8948937	NK_H8_118	Kp1	SL17	2018	Human	Blood	SAMEA111533205
KP_NORM_BLD_116570	Kp1	SL1373	2015	Human	Blood	SAMEA8948938	NK_H8_121	Kp1	SL17	2018	Human	Blood	SAMEA113605334
KP_NORM_BLD_117477	Kp1	SL661	2015	Human	Blood	SAMEA8948948	NK_H9_001	Kp1	SL882	2017	Human	Blood	SAMEA113605336
KP_NORM_BLD_12850	Kp1	SL37	2001	Human	Blood	SAMEA8948284	NK_H9_003	Kp1	SL10508	2017	Human	Blood	SAMEA113605338
KP_NORM_BLD_12934	Kp3	SL1875	2001	Human	Blood	SAMEA8948295	NK_H9_006	Kp1	SL3804	2017	Human	Blood	SAMEA113605341
KP_NORM_BLD_13123	Kp1	SL188	2001	Human	Blood	SAMEA8948308	T7-007	Kp1	SL3010	2015	Human	Fecal	SAMEA7773558
KP_NORM_BLD_13127	Kp3	SL641	2001	Human	Blood	SAMEA8948311	T7-010	Kp1	SL3010	2015	Human	Fecal	SAMEA7773561
KP_NORM_BLD_13129	Kp1	SL6	2001	Human	Blood	SAMEA8948313	T7-011	Kp1	SL10828	2015	Human	Fecal	SAMEA7773562
KP_NORM_BLD_13372	Kp1	SL322	2001	Human	Blood	SAMEA8948321	T7-012	Kp1	SL17	2015	Human	Fecal	SAMEA7773563
KP_NORM_BLD_13375	Kp1	SL15	2001	Human	Blood	SAMEA8948324	T7-013	Kp1	SL45	2015	Human	Fecal	SAMEA7773564
KP_NORM_BLD_13377	Kp4	SL11781	2001	Human	Blood	SAMEA8948326	T7-019	Kp1	SL3010	2015	Human	Fecal	SAMEA7773569
KP_NORM_BLD_13379	Kp1	SL10	2001	Human	Blood	SAMEA8948328	T7-020	Kp1	SL17	2015	Human	Fecal	SAMEA7773570
KP_NORM_BLD_13380	Kp1	SL76	2001	Human	Blood	SAMEA8948329	T7-032	Kp3	SL10829	2015	Human	Fecal	SAMEA7773582
KP_NORM_BLD_13382	Kp1	SL3010	2001	Human	Blood	SAMEA8948331	T7-034	Kp1	SL10830	2015	Human	Fecal	SAMEA7773584
KP_NORM_BLD_13383	Kp1	SL3370	2001	Human	Blood	SAMEA8948332	T7-036	Kp1	SL716	2015	Human	Fecal	SAMEA7773586
KP_NORM_BLD_1339	Kp3	SL595	2005	Human	Blood	SAMEA8948198	T7-050	Kp3	SL10056	2015	Human	Fecal	SAMEA7773600
KP_NORM_BLD_1347	Kp1	SL11065	2005	Human	Blood	SAMEA8948203	T7-051	Kp1	SL1799	2015	Human	Fecal	SAMEA7773601
KP_NORM_BLD_1348	Kp3	SL1875	2005	Human	Blood	SAMEA8948204	T7-072	Kp2	SL10771	2015	Human	Fecal	SAMEA7773622
KP_NORM_BLD_13534	Kp1	SL1082	2001	Human	Blood	SAMEA8948337	T7-078	Kp1	SL3583	2015	Human	Fecal	SAMEA7773628
KP_NORM_BLD_13738	Kp1	SL27	2001	Human	Blood	SAMEA8948345	T7-085	Kp1	SL34	2015	Human	Fecal	SAMEA7773635
KP_NORM_BLD_13748	Kp1	SL6	2001	Human	Blood	SAMEA8948351	T7-089	Kp2	SL10786	2015	Human	Fecal	SAMEA7773639
KP_NORM_BLD_13757	Kp4	SL1535	2001	Human	Blood	SAMEA8948355	T7-096	Kp1	SL2791	2015	Human	Fecal	SAMEA7773646
KP_NORM_BLD_13880	Kp1	SL453	2001	Human	Blood	SAMEA8948358	T7-097	Kp1	SL461	2015	Human	Fecal	SAMEA7773647
KP_NORM_BLD_13886	Kp3	SL1169	2001	Human	Blood	SAMEA8948361	T7-103	Kp1	SL2004	2015	Human	Fecal	SAMEA7773653
KP_NORM_BLD_13889	Kp1	SL416	2001	Human	Blood	SAMEA8948364	T7-105	Kp3	SL10835	2015	Human	Fecal	SAMEA7773655
KP_NORM_BLD_14164	Kp4	SL384	2001	Human	Blood	SAMEA8948377	T7-115	Kp1	SL17	2015	Human	Fecal	SAMEA7773665
KP_NORM_BLD_14177	Kp1	SL3010	2001	Human	Blood	SAMEA8948389	T7-125	Kp1	SL10838	2015	Human	Fecal	SAMEA7773675
KP_NORM_BLD_14179	Kp1	SL359	2001	Human	Blood	SAMEA8948391	T7-129	Kp1	SL867	2015	Human	Fecal	SAMEA7773679
KP_NORM_BLD_14222	Kp1	SL30	2001	Human	Blood	SAMEA8948393	T7-133	Kp3	SL681	2015	Human	Fecal	SAMEA7773683
KP_NORM_BLD_1692	Kp1	SL10375	2005	Human	Blood	SAMEA8948219	T7-141	Kp1	SL37	2015	Human	Fecal	SAMEA7773690
KP_NORM_BLD_1883	Kp1	SL3010	2005	Human	Blood	SAMEA8948228	T7-146	Kp1	SL10334	2015	Human	Fecal	SAMEA7773695
KP_NORM_BLD_2005_1167	Kp1	SL35	2005	Human	Blood	SAMEA8948196	T7-153	Kp1	SL461	2015	Human	Fecal	SAMEA7773702
KP_NORM_BLD_2006_36504	Kp1	SL22	2006	Human	Blood	SAMEA8948395	T7-158	Kp1	SL10799	2015	Human	Fecal	SAMEA7773707
KP_NORM_BLD_2006_37102	Kp1	SL874	2006	Human	Blood	SAMEA8948396	T7-160	Kp3	SL2386	2015	Human	Fecal	SAMEA7773709
KP_NORM_BLD_2007_46696	Kp1	SL10971	2007	Human	Blood	SAMEA8948397	T7-165	Kp3	SL250	2015	Human	Fecal	SAMEA7773714
KP_NORM_BLD_2007_48102	Kp1	SL45	2007	Human	Blood	SAMEA115163002	T7-179	Kp3	SL10840	2015	Human	Fecal	SAMEA7773728
KP_NORM_BLD_2007_48114	Kp1	SL111	2007	Human	Blood	SAMEA115163003	T7-180	Kp1	SL152	2015	Human	Fecal	SAMEA7773729
KP_NORM_BLD_2008_54367	Kp1	SL231	2008	Human	Blood	SAMEA8948402	T7-181	Kp1	SL1189	2015	Human	Fecal	SAMEA7773730
KP_NORM_BLD_2008_54823	Kp1	SL258	2008	Human	Blood	SAMEA115163004	T7-185	Kp1	SL314	2015	Human	Fecal	SAMEA7773734
KP_NORM_BLD_2008_54824	Kp1	SL397	2008	Human	Blood	SAMEA115374654	T7-189	Kp1	SL3010	2015	Human	Fecal	SAMEA7773738
KP_NORM_BLD_2008_55014	Kp1	SL10194	2008	Human	Blood	SAMEA8948403	T7-200	Kp1	SL3010	2015	Human	Fecal	SAMEA7773749
KP_NORM_BLD_2008_55153	Kp1	SL27	2008	Human	Blood	SAMEA8948404	T7-209	Kp1	SL10332	2015	Human	Fecal	SAMEA7773758
KP_NORM_BLD_2009_61298	Kp1	SL14	2009	Human	Blood	SAMEA8948470	T7-211	Kp1	SL29	2015	Human	Fecal	SAMEA7773760
KP_NORM_BLD_2009_61895	Kp1	SL15	2009	Human	Blood	SAMEA5063293	T7-213	Kp1	SL1432	2015	Human	Fecal	SAMEA7773762
KP_NORM_BLD_2009_62172	Kp1	SL231	2009	Human	Blood	SAMEA8948510	T7-220	Kp3	SL357	2015	Human	Fecal	SAMEA7773769
KP_NORM_BLD_2009_62777	Kp1	SL14	2009	Human	Blood	SAMEA8948556	T7-225	Kp1	SL36	2015	Human	Fecal	SAMEA7773774
KP_NORM_BLD_2009_63475	Kp1	SL35	2009	Human	Blood	SAMEA8948585	T7-234	Kp1	SL465	2015	Human	Fecal	SAMEA7773783
KP_NORM_BLD_2009_63807	Kp1	SL17	2009	Human	Blood	SAMEA8948592	T7-240	Kp1	SL45	2015	Human	Fecal	SAMEA7773789
KP_NORM_BLD_2010_70236	Kp1	SL70	2010	Human	Blood	SAMEA8948610	T7-242	Kp1	SL3010	2015	Human	Fecal	SAMEA7773791
KP_NORM_BLD_2010_70364	Kp1	SL10972	2010	Human	Blood	SAMEA8948611	T7-259	Kp1	SL258	2015	Human	Fecal	SAMEA7773808
KP_NORM_BLD_2010_71621	Kp1	SL258	2010	Human	Blood	SAMEA8602826	T7-262	Kp2	SL10199	2015	Human	Fecal	SAMEA7773811
KP_NORM_BLD_2011_74838	Kp1	SL383	2011	Human	Blood	SAMEA8948626	T7-264	Kp1	SL10809	2015	Human	Fecal	SAMEA7773813
KP_NORM_BLD_2011_75117	Kp1	SL45	2011	Human	Blood	SAMEA8948630	T7-266	Kp4	SL10777	2015	Human	Fecal	SAMEA7773815
KP_NORM_BLD_2011_75461	Kp1	SL37	2011	Human	Blood	SAMEA8948633	T7-267	Kp3	SL1848	2015	Human	Fecal	SAMEA7773816
KP_NORM_BLD_2012_85054	Kp1	SL307	2012	Human	Blood	SAMEA4724625	T7-273	Kp1	SL10393	2015	Human	Fecal	SAMEA7773822
KP_NORM_BLD_2012_85408	Kp1	SL258	2012	Human	Blood	SAMEA9972033	T7-286	Kp3	SL3937	2015	Human	Fecal	SAMEA7773835
KP_NORM_BLD_2012_86925	Kp1	SL10395	2012	Human	Blood	SAMEA8948655	T7-289	Kp1	SL277	2015	Human	Fecal	SAMEA7773837
KP_NORM_BLD_2012_86962	Kp1	SL70	2012	Human	Blood	SAMEA8948657	T7-290	Kp1	SL35	2015	Human	Fecal	SAMEA7773838
KP_NORM_BLD_2013_88977	Kp1	SL258	2013	Human	Blood	SAMEA9972036	T7-292	Kp1	SL35	2015	Human	Fecal	SAMEA7773840
KP_NORM_BLD_2013_90851	Kp1	SL427	2013	Human	Blood	SAMEA8948660	T7-296	Kp1	SL3010	2015	Human	Fecal	SAMEA7773844
KP_NORM_BLD_2013_91198	Kp1	SL231	2013	Human	Blood	SAMEA8948662	T7-311	Kp3	SL641	2015	Human	Fecal	SAMEA7773859
KP_NORM_BLD_2013_91444	Kp1	SL258	2013	Human	Blood	SAMEA8948664	T7-326	Kp1	SL661	2015	Human	Fecal	SAMEA7773874
KP_NORM_BLD_2013_92320	Kp1	SL10147	2013	Human	Blood	SAMEA8948666	T7-333	Kp1	SL2004	2015	Human	Fecal	SAMEA7773881
KP_NORM_BLD_2013_92328	Kp1	SL6	2013	Human	Blood	SAMEA8948669	T7-335	Kp3	SL10851	2015	Human	Fecal	SAMEA7773883
KP_NORM_BLD_2014_100833	Kp1	SL258	2014	Human	Blood	SAMEA8948698	T7-349	Kp1	SL3010	2015	Human	Fecal	SAMEA7773896
KP_NORM_BLD_2014_100848	Kp1	SL307	2014	Human	Blood	SAMEA4724627	T7-365	Kp1	SL17	2015	Human	Fecal	SAMEA7773911
KP_NORM_BLD_2014_101320	Kp1	SL307	2014	Human	Blood	SAMEA4724630	T7-378	Kp1	SL34	2015	Human	Fecal	SAMEA7773924
KP_NORM_BLD_2014_101821	Kp1	SL147	2014	Human	Blood	SAMEA8948701	T7-393	Kp1	SL10221	2015	Human	Fecal	SAMEA7773938
KP_NORM_BLD_2014_102946	Kp1	SL15	2014	Human	Blood	SAMEA5063297	T7-394	Kp1	SL2042	2015	Human	Fecal	SAMEA7773939
KP_NORM_BLD_2014_103381	Kp1	SL307	2014	Human	Blood	SAMEA4724634	T7-398	Kp1	SL359	2015	Human	Fecal	SAMEA7773943
KP_NORM_BLD_2014_104014	Kp1	SL15	2014	Human	Blood	SAMEA5063299	T7-402	Kp3	SL10253	2015	Human	Fecal	SAMEA7773946
KP_NORM_BLD_2014_104302	Kp1	SL14	2014	Human	Blood	SAMEA8948707	T7-410	Kp1	SL461	2015	Human	Fecal	SAMEA7773954
KP_NORM_BLD_2014_96855	Kp1	SL420	2014	Human	Blood	SAMEA8602828	T7-414	Kp1	SL34	2015	Human	Fecal	SAMEA7773958
KP_NORM_BLD_2015_108843	Kp1	SL14	2015	Human	Blood	SAMEA9972038	T7-424	Kp3	SL1562	2015	Human	Fecal	SAMEA7773968
KP_NORM_BLD_2015_111587	Kp1	SL258	2015	Human	Blood	SAMEA8948739	T7-425	Kp3	SL641	2015	Human	Fecal	SAMEA7773969
KP_NORM_BLD_2015_112126	Kp1	SL15	2015	Human	Blood	SAMEA5063300	T7-439	Kp1	SL3010	2015	Human	Fecal	SAMEA7773982
KP_NORM_BLD_2015_115359	Kp1	SL45	2015	Human	Blood	SAMEA8602827	T7-442	Kp1	SL23	2015	Human	Fecal	SAMEA7773985
KP_NORM_BLD_2247	Kp1	SL17	2005	Human	Blood	SAMEA8948234	T7-445	Kp1	SL1145	2015	Human	Fecal	SAMEA7773988
KP_NORM_BLD_225	Kp1	SL11207	2005	Human	Blood	SAMEA8948154	T7-457	Kp1	SL10393	2015	Human	Fecal	SAMEA7774000
KP_NORM_BLD_2254	Kp1	SL152	2005	Human	Blood	SAMEA8948238	T7-460	Kp1	SL1496	2015	Human	Fecal	SAMEA7774003
KP_NORM_BLD_228	Kp1	SL76	2005	Human	Blood	SAMEA8948156	T7-472	Kp3	SL10427	2016	Human	Fecal	SAMEA7774015
KP_NORM_BLD_231	Kp1	SL416	2005	Human	Blood	SAMEA8948158	T7-475	Kp1	SL151	2016	Human	Fecal	SAMEA7774018
KP_NORM_BLD_2395	Kp1	SL107	2005	Human	Blood	SAMEA8948241	T7-482	Kp3	SL1423	2016	Human	Fecal	SAMEA7774025
KP_NORM_BLD_2497	Kp1	SL870	2005	Human	Blood	SAMEA8948250	T7-488	Kp1	SL2042	2016	Human	Fecal	SAMEA7774031
KP_NORM_BLD_2508	Kp1	SL10300	2005	Human	Blood	SAMEA8948256	T7-493	Kp3	SL10181	2016	Human	Fecal	SAMEA7774035
KP_NORM_BLD_2799	Kp1	SL35	2005	Human	Blood	SAMEA8948264	KP_NORM_URN_106487	Kp3	SL1456	2015	Human	Urine	SAMEA114492505
KP_NORM_BLD_3074	Kp1	SL134	2005	Human	Blood	SAMEA8948270	KP_NORM_URN_106847	Kp1	SL17	2015	Human	Urine	SAMEA114492509
KP_NORM_BLD_60429	Kp1	SL3010	2009	Human	Blood	SAMEA8948414	KP_NORM_URN_107976	Kp1	SL3010	2015	Human	Urine	SAMEA115163005
KP_NORM_BLD_60562	Kp3	SL595	2009	Human	Blood	SAMEA8948424	KP_NORM_URN_109342	Kp3	SL10846	2015	Human	Urine	SAMEA114492542
KP_NORM_BLD_60723	Kp1	SL11107	2009	Human	Blood	SAMEA8948430	KP_NORM_URN_113027	Kp1	SL528	2015	Human	Urine	SAMEA114492575
KP_NORM_BLD_60778	Kp3	SL1478	2009	Human	Blood	SAMEA8948433	KP_NORM_URN_114421	Kp1	SL13	2015	Human	Urine	SAMEA114492582
KP_NORM_BLD_60803	Kp2	SL11785	2009	Human	Blood	SAMEA8948436	KP_NORM_URN_2009_57401	Kp1	SL10970	2009	Human	Urine	SAMEA8948408
KP_NORM_BLD_60874	Kp1	SL10874	2009	Human	Blood	SAMEA8948439	KP_NORM_URN_2012_81416	Kp1	SL10970	2012	Human	Urine	SAMEA8948642
KP_NORM_BLD_61273	Kp2	SL526	2009	Human	Blood	SAMEA8948460	KP_NORM_URN_2012_81428	Kp1	SL307	2012	Human	Urine	SAMEA4724642
KP_NORM_BLD_61295	Kp1	SL323	2009	Human	Blood	SAMEA8948468	KP_NORM_URN_2013_95439	Kp1	SL147	2013	Human	Urine	SAMEA8948675
KP_NORM_BLD_61439	Kp1	SL461	2009	Human	Blood	SAMEA8948474	KP_NORM_URN_2013_95788	Kp1	SL258	2013	Human	Urine	SAMEA8948679
KP_NORM_BLD_61444	Kp3	SL11203	2009	Human	Blood	SAMEA8948476	KP_NORM_URN_2013_95819	Kp1	SL39	2013	Human	Urine	SAMEA8948680
KP_NORM_BLD_61459	Kp1	SL38	2009	Human	Blood	SAMEA8948481	KP_NORM_URN_2013_95951	Kp1	SL48	2013	Human	Urine	SAMEA8948682
KP_NORM_BLD_61886	Kp3	SL11133	2009	Human	Blood	SAMEA8948497	KP_NORM_URN_2013_96200	Kp1	SL307	2013	Human	Urine	SAMEA4745130
KP_NORM_BLD_61894	Kp1	SL12	2009	Human	Blood	SAMEA8948500	KP_NORM_URN_2014_101205	Kp1	SL45	2014	Human	Urine	SAMEA8948699
KP_NORM_BLD_62164	Kp1	SL43	2009	Human	Blood	SAMEA8948506	KP_NORM_URN_2014_96612	Kp1	SL258	2014	Human	Urine	SAMEA8948685
KP_NORM_BLD_62202	Kp1	SL1035	2009	Human	Blood	SAMEA8948518	KP_NORM_URN_2014_97392	Kp1	SL48	2014	Human	Urine	SAMEA8948687
KP_NORM_BLD_62486	Kp3	SL10763	2009	Human	Blood	SAMEA8948532	KP_NORM_URN_2014_97411	Kp1	SL416	2014	Human	Urine	SAMEA8948688
KP_NORM_BLD_62504	Kp1	SL359	2009	Human	Blood	SAMEA8948538	KP_NORM_URN_2014_97671	Kp1	SL45	2014	Human	Urine	SAMEA8948689
KP_NORM_BLD_62512	Kp3	SL11119	2009	Human	Blood	SAMEA8948541	KP_NORM_URN_2014_97677	Kp1	SL11073	2014	Human	Urine	SAMEA8948690
KP_NORM_BLD_62638	Kp1	SL432	2009	Human	Blood	SAMEA8948543	KP_NORM_URN_2014_99206	Kp1	SL17	2014	Human	Urine	SAMEA8948693
KP_NORM_BLD_62649	Kp1	SL152	2009	Human	Blood	SAMEA8948546	KP_NORM_URN_2015_107836	Kp1	SL198	2015	Human	Urine	SAMEA8948712
KP_NORM_BLD_62912	Kp1	SL881	2009	Human	Blood	SAMEA8948557	KP_NORM_URN_2015_107962	Kp1	SL10016	2015	Human	Urine	SAMEA8948713
KP_NORM_BLD_62914	Kp1	SL967	2009	Human	Blood	SAMEA8948558	KP_NORM_URN_2015_109056	Kp1	SL37	2015	Human	Urine	SAMEA8948722
KP_NORM_BLD_62920	Kp1	SL11106	2009	Human	Blood	SAMEA8948561	KP_NORM_URN_2015_109238	Kp1	SL10975	2015	Human	Urine	SAMEA8948724
KP_NORM_BLD_62922	Kp1	SL11105	2009	Human	Blood	SAMEA8948562	KP_NORM_URN_2015_109358	Kp1	SL611	2015	Human	Urine	SAMEA8948725
KP_NORM_BLD_62926	Kp1	SL294	2009	Human	Blood	SAMEA8948564	KP_NORM_URN_2015_109604	Kp1	SL307	2015	Human	Urine	SAMEA4724651
KP_NORM_BLD_62934	Kp1	SL1436	2009	Human	Blood	SAMEA8948568	KP_NORM_URN_56534	Kp1	SL2791	2009	Human	Urine	SAMEA115163006
KP_NORM_BLD_63395	Kp1	SL3010	2009	Human	Blood	SAMEA8948584	KP_NORM_URN_56668	Kp1	SL17	2009	Human	Urine	SAMEA115163007
KP_NORM_BLD_63801	Kp1	SL17	2009	Human	Blood	SAMEA8948589	KP_NORM_URN_56847	Kp3	SL595	2009	Human	Urine	SAMEA115374676
KP_NORM_BLD_63815	Kp1	SL10899	2009	Human	Blood	SAMEA8948595	KP_NORM_URN_57002	Kp1	SL1626	2009	Human	Urine	SAMEA115163008
KP_NORM_BLD_63832	Kp1	SL86	2009	Human	Blood	SAMEA8948601	KP_NORM_URN_57023	Kp3	SL641	2009	Human	Urine	SAMEA115163009
KP_NORM_BLD_63839	Kp1	SL322	2009	Human	Blood	SAMEA8948604	KP_NORM_URN_57042	Kp1	SL25	2009	Human	Urine	SAMEA115163010
KP_NORM_BLD_63842	Kp1	SL152	2009	Human	Blood	SAMEA8948605	KP_NORM_URN_57225	Kp1	SL3010	2009	Human	Urine	SAMEA115163011
KP_NORM_BLD_70	Kp1	SL11089	2005	Human	Blood	SAMEA8948152	KP_NORM_URN_57409	Kp1	SL3591	2009	Human	Urine	SAMEA115163012
NK_H1_001	Kp1	SL188	2017	Human	Blood	SAMEA113604969	KP_NORM_URN_57447	Kp3	SL11768	2009	Human	Urine	SAMEA115163013
NK_H1_002	Kp1	SL35	2017	Human	Blood	SAMEA113604970	KP_NORM_URN_57455	Kp3	SL11769	2009	Human	Urine	SAMEA115163014
NK_H1_011	Kp1	SL3583	2017	Human	Blood	SAMEA113604977	KP_NORM_URN_57695	Kp1	SL45	2009	Human	Urine	SAMEA115163015
NK_H1_012	Kp1	SL1966	2017	Human	Blood	SAMEA113604978	KP_NORM_URN_57979	Kp1	SL215	2009	Human	Urine	SAMEA115163016
NK_H1_037	Kp1	SL10019	2017	Human	Blood	SAMEA113604995	KP_NORM_URN_58012	Kp3	SL2594	2009	Human	Urine	SAMEA115163017
NK_H1_040	Kp1	SL551	2017	Human	Blood	SAMEA113604997	KP_NORM_URN_58233	Kp1	SL3747	2009	Human	Urine	SAMEA115163018
NK_H1_041	Kp1	SL107	2017	Human	Blood	SAMEA113604998	KP_NORM_URN_58251	Kp1	SL322	2009	Human	Urine	SAMEA115163019
NK_H1_044	Kp3	SL10403	2017	Human	Blood	SAMEA113605000	KP_NORM_URN_58261	Kp1	SL15	2009	Human	Urine	SAMEA115163020
NK_H1_050	Kp1	SL1997	2017	Human	Blood	SAMEA113605006	KP_NORM_URN_58288	Kp1	SL45	2009	Human	Urine	SAMEA115163023
NK_H1_059	Kp3	SL595	2017	Human	Blood	SAMEA113605013	KP_NORM_URN_58342	Kp1	SL10	2009	Human	Urine	SAMEA115163021
NK_H1_074	Kp1	SL461	2018	Human	Blood	SAMEA113605027	KP_NORM_URN_58394	Kp1	SL4	2009	Human	Urine	SAMEA115163022
NK_H1_084	Kp1	SL35	2018	Human	Blood	SAMEA113605036	KGMAR-2016-1076	Kp1	SL22	2016	M. edulis		SAMN13382579
NK_H1_091	Kp1	SL882	2018	Human	Blood	SAMEA113605042	KGMAR-2016-1178	Kp1	SL11219	2016	M. edulis		SAMN13382581
NK_H1_098	Kp1	SL383	2018	Human	Blood	SAMEA113605049	KGMAR-2016-1198	Kp1	SL37	2016	M. edulis		SAMN13382582
NK_H1_109	Kp1	SL278	2018	Human	Blood	SAMEA113605055	KGMAR-2016-1200	Kp1	SL25	2016	M. edulis		SAMN13382583
NK_H1_111	Kp1	SL461	2018	Human	Blood	SAMEA113605057	KGMAR-2016-1396	Kp1	SL3010	2016	M. edulis		SAMN13382596
NK_H10_003	Kp1	SL611	2017	Human	Blood	SAMEA113605348	KGMAR-2016-1397	Kp1	SL872	2016	M. edulis		SAMN13382597
NK_H11_006	Kp1	SL23	2017	Human	Blood	SAMEA113605358	KGMAR-2016-1400	Kp1	SL1035	2016	M. edulis		SAMEA7293484
NK_H11_013	Kp1	SL14	2018	Human	Blood	SAMEA113605363	KGMAR-2016-563	Kp1	SL383	2016	M. edulis		SAMN13382564
NK_H12_018	Kp1	SL107	2017	Human	Blood	SAMEA113605380	KGMAR-2016-733	Kp1	SL252	2016	M. edulis		SAMN13382572
NK_H12_023	Kp1	SL2042	2017	Human	Blood	SAMEA113605732	KGMAR-2019-1417-2	Kp1	SL3157	2019	M. edulis		SAMN17766468
NK_H12_028	Kp1	SL107	2017	Human	Blood	SAMEA113605386	KGMAR-2019-1434	Kp6	SL11028	2019	M. edulis		SAMN17766469
NK_H12_034	Kp1	SL25	2017	Human	Blood	SAMEA113605391	KGMAR-2019-1459	Kp1	SL922	2019	M. edulis		SAMN17766472
NK_H12_035	Kp1	SL23	2017	Human	Blood	SAMEA113605392	KGMAR-2019-1497	Kp1	SL45	2019	M. edulis		SAMN17766475
NK_H12_038	Kp1	SL2042	2017	Human	Blood	SAMEA113605743	KGMAR-2019-1764	Kp1	SL292	2019	M. edulis		SAMN17766478
NK_H12_041	Kp1	SL1626	2017	Human	Blood	SAMEA113605398	KGMAR-2019-1792	Kp1	SL314	2019	M. edulis		SAMN17766482
NK_H12_048	Kp1	SL3647	2017	Human	Blood	SAMEA113605405	KGMAR-2019-2186	Kp1	SL3676	2019	M. edulis		SAMN17766494
NK_H12_053	Kp1	SL1269	2017	Human	Blood	SAMEA113605410	KGMAR-2020-428	Kp1	SL34	2020	M. edulis		SAMN17766507
NK_H12_057	Kp4	SL1191	2018	Human	Blood	SAMEA113605414	KGMAR-2020-440	Kp1	SL1877	2020	M. edulis		SAMN17766508
NK_H12_062	Kp1	SL10174	2018	Human	Blood	SAMEA113605417	KGVETS-2019-01-1336	Kp1	SL10076	2019	Pig	Cecal	SAMN28106918
NK_H12_063	Kp1	SL101	2018	Human	Blood	SAMEA113605418	KGVETS-2019-01-1378	Kp1	SL221	2019	Pig	Cecal	SAMN28626224
NK_H12_068	Kp3	SL11161	2017	Human	Blood	SAMEA113605421	KGVETS-2019-01-1380	Kp3	SL10309	2019	Pig	Cecal	SAMN28626226
NK_H12_086	Kp1	SL15	2018	Human	Blood	SAMEA113605435	KGVETS-2019-01-1382	Kp1	SL113	2019	Pig	Cecal	SAMN28626228
NK_H12_100	Kp3	SL10403	2018	Human	Blood	SAMEA113605447	KGVETS-2019-01-1759	Kp1	SL107	2019	Pig	Cecal	SAMN28106919
NK_H13_003	Kp2	SL11303	2017	Human	Blood	SAMEA113605461	KGVETS-2019-01-1760	Kp1	SL107	2019	Pig	Cecal	SAMN28626241
NK_H14_001	Kp1	SL17	2017	Human	Blood	SAMEA111533192	KGVETS-2019-01-1761	Kp1	SL2411	2019	Pig	Cecal	SAMN28626242
NK_H14_010	Kp1	SL107	2017	Human	Blood	SAMEA113605482	KGVETS-2019-01-1806	Kp1	SL37	2019	Pig	Cecal	SAMN28106920
NK_H14_015	Kp4	SL10598	2017	Human	Blood	SAMEA113605487	KGVETS-2019-01-1844	Kp1	SL113	2019	Pig	Cecal	SAMN28626246
NK_H14_033	Kp3	SL250	2017	Human	Blood	SAMEA113605504	KGVETS-2019-01-1961	Kp1	SL10300	2019	Pig	Cecal	SAMN28106921
NK_H14_036	Kp1	SL45	2017	Human	Blood	SAMEA113605506	KGVETS-2019-01-2116	Kp3	SL2594	2019	Pig	Cecal	SAMN28626258
NK_H14_037	Kp1	SL186	2017	Human	Blood	SAMEA113605507	KGVETS-2019-01-2201	Kp1	SL10130	2019	Pig	Cecal	SAMN28626263
NK_H14_042	Kp3	SL10368	2017	Human	Blood	SAMEA113605512	KGVETS-2019-01-2303	Kp4	SL138	2019	Pig	Cecal	SAMN28626267
NK_H14_050	Kp1	SL461	2017	Human	Blood	SAMEA113605519	KGVETS-2019-01-2870	Kp1	SL10880	2019	Pig	Cecal	SAMN28626275
NK_H14_057	Kp1	SL49	2017	Human	Blood	SAMEA113605525	KGVETS-2019-01-2871	Kp1	SL200	2019	Pig	Cecal	SAMN28626276
NK_H14_058	Kp4	SL11296	2017	Human	Blood	SAMEA113605526	KGVETS-2019-01-3127	Kp1	SL1626	2019	Pig	Cecal	SAMN28626287
NK_H14_063	Kp1	SL1785	2018	Human	Blood	SAMEA113605530	KGVETS-2019-01-3158	Kp3	SL10007	2019	Pig	Cecal	SAMN28626290
NK_H15_001	Kp3	SL10763	2017	Human	Blood	SAMEA113605554	KGVETS-2019-01-3162	Kp1	SL290	2019	Pig	Cecal	SAMN28106922
NK_H15_002	Kp1	SL25	2017	Human	Blood	SAMEA113605555	KGVETS-2019-01-3185	Kp1	SL10878	2019	Pig	Cecal	SAMN28106923
NK_H15_014	Kp3	SL1562	2017	Human	Blood	SAMEA113605566	KGVETS-2019-01-3187	Kp1	SL37	2019	Pig	Cecal	SAMN28626296
NK_H15_021	Kp1	SL101	2017	Human	Blood	SAMEA113605573	KGVETS-2019-01-326	Kp1	SL35	2019	Pig	Cecal	SAMN28106924
NK_H15_022	Kp1	SL86	2017	Human	Blood	SAMEA113605574	KGVETS-2019-01-376	Kp1	SL1198	2019	Pig	Cecal	SAMN28626299
NK_H15_025	Kp3	SL641	2017	Human	Blood	SAMEA113605577	KGVETS-2019-01-3793	Kp1	SL301	2019	Pig	Cecal	SAMN28626300
NK_H15_032	Kp1	SL133	2017	Human	Blood	SAMEA113605583	KGVETS-2019-01-4118	Kp1	SL116	2019	Pig	Cecal	SAMN28626304
NK_H15_042	Kp1	SL3370	2017	Human	Blood	SAMEA113605590	KGVETS-2019-01-4380	Kp1	SL10572	2019	Pig	Cecal	SAMN28106925
NK_H15_047	Kp1	SL2004	2017	Human	Blood	SAMEA113605594	KGVETS-2019-01-4407	Kp1	SL10882	2019	Pig	Cecal	SAMN28626316
NK_H15_048	Kp1	SL107	2017	Human	Blood	SAMEA113605595	KGVETS-2019-01-4548	Kp1	SL37	2019	Pig	Cecal	SAMN28626318
NK_H15_056	Kp1	SL66	2018	Human	Blood	SAMEA113605602	KGVETS-2019-01-4637	Kp1	SL10885	2019	Pig	Cecal	SAMN28106926
NK_H15_060	Kp1	SL10175	2018	Human	Blood	SAMEA113605606	KGVETS-2019-01-4639	Kp1	SL10176	2019	Pig	Cecal	SAMN28626323
NK_H16_005	Kp1	SL107	2017	Human	Blood	SAMEA113605622	KGVETS-2019-01-476	Kp1	SL60	2019	Pig	Cecal	SAMN28626329
NK_H16_012	Kp1	SL152	2017	Human	Blood	SAMEA113605629	KGVETS-2019-01-4908	Kp1	SL10886	2019	Pig	Cecal	SAMN28626331
NK_H16_016	Kp1	SL10215	2018	Human	Blood	SAMEA113605633	KGVETS-2019-01-4937	Kp1	SL3607	2019	Pig	Cecal	SAMN28106927
NK_H16_021	Kp1	SL380	2018	Human	Blood	SAMEA113605638	KGVETS-2019-01-5108	Kp1	SL1628	2019	Pig	Cecal	SAMN28626334
NK_H16_023	Kp3	SL641	2017	Human	Blood	SAMEA113605640	KGVETS-2019-01-587	Kp1	SL44	2019	Pig	Cecal	SAMN28626335
NK_H17_002	Kp3	SL919	2018	Human	Blood	SAMEA113605642	KGVETS-2019-01-604	Kp1	SL629	2019	Pig	Cecal	SAMN28626339
NK_H17_017	Kp1	SL11298	2017	Human	Blood	SAMEA113605655	KGVETS-2019-01-897	Kp1	SL661	2019	Pig	Cecal	SAMN28106928
NK_H17_018	Kp1	SL1626	2017	Human	Blood	SAMEA113605656	KGVETS-2019-01-901	Kp1	SL3050	2019	Pig	Cecal	SAMN28626347
NK_H17_023	Kp1	SL1307	2017	Human	Blood	SAMEA113605661	KGVETS-2019-01-902	Kp1	SL45	2019	Pig	Cecal	SAMN28106929
NK_H17_034	Kp1	SL359	2017	Human	Blood	SAMEA113605670	KGMAR-2019-1766	Kp1	SL17	2019	Seawater		SAMN17766480
NK_H17_042	Kp1	SL247	2017	Human	Blood	SAMEA113605677	KGMAR-2019-400-2	Kp1	SL37	2019	Seawater		SAMN17766463
NK_H18_003	Kp1	SL10127	2017	Human	Blood	SAMEA113605681	KGMAR-2019-604	Kp1	SL111	2019	Seawater		SAMN17766464
NK_H18_005	Kp1	SL297	2017	Human	Blood	SAMEA113605683	KGVET-2018-01-4546	Kp1	SL290	2018	Turkey	Air sac	SAMN32747447
NK_H18_019	Kp1	SL3639	2017	Human	Blood	SAMEA113605696	2018-01-1001	Kp1	SL35	2018	Turkey	Cecal	SAMEA8399237
NK_H18_026	Kp1	SL391	2017	Human	Blood	SAMEA113605703	2018-01-1024	Kp1	SL10868	2018	Turkey	caecal	SAMEA8399241
NK_H18_038	Kp1	SL280	2017	Human	Blood	SAMEA113605713	2018-01-1097	Kp1	SL290	2018	Turkey	Cecal	SAMEA8399243
NK_H18_050	Kp1	SL200	2018	Human	Blood	SAMEA113605724	2018-01-1439	Kp1	SL1229	2018	Turkey	Cecal	SAMEA8399260
NK_H18_054	Kp1	SL11135	2018	Human	Blood	SAMEA113605728	2018-01-1584	Kp1	SL35	2018	Turkey	Cecal	SAMEA8399265
NK_H18_072	Kp1	SL611	2018	Human	Blood	SAMEA113605745	2018-01-2085	Kp1	SL35	2018	Turkey	Cecal	SAMEA8399289
NK_H18_073	Kp1	SL25	2018	Human	Blood	SAMEA113605746	2018-01-2485	Kp1	SL290	2018	Turkey	Cecal	SAMEA8399314
NK_H18_074	Kp1	SL3682	2018	Human	Blood	SAMEA113605747	2018-01-2640	Kp1	SL37	2018	Turkey	Cecal	SAMEA8399324
NK_H19_011	Kp1	SL66	2017	Human	Blood	SAMEA113605759	2018-01-2730	Kp1	SL37	2018	Turkey	Cecal	SAMEA8399332
NK_H19_015	Kp1	SL997	2017	Human	Blood	SAMEA113605762	2018-01-2860	Kp1	SL528	2018	Turkey	Cecal	SAMEA8399341
NK_H2_007	Kp1	SL314	2017	Human	Blood	SAMEA113605072	2018-01-3030	Kp1	SL35	2018	Turkey	Cecal	SAMEA8399352
NK_H2_019	Kp3	SL1562	2017	Human	Blood	SAMEA113605084	2018-01-3094	Kp1	SL1944	2018	Turkey	Cecal	SAMEA8399355
NK_H2_020	Kp1	SL45	2018	Human	Blood	SAMEA113605085	2018-01-3218	Kp1	SL10503	2018	Turkey	Cecal	SAMEA8399363
NK_H2_021	Kp1	SL10371	2018	Human	Blood	SAMEA113605086	2018-01-3465	Kp1	SL10475	2018	Turkey	Cecal	SAMEA8399380
NK_H20_007	Kp1	SL1629	2017	Human	Blood	SAMEA113605777	2018-01-4107	Kp1	SL35	2018	Turkey	Cecal	SAMEA8399427
NK_H20_010	Kp1	SL3614	2017	Human	Blood	SAMEA113605780	2018-01-4735	Kp1	SL37	2018	Turkey	Cecal	SAMEA8399450
NK_H20_015	Kp1	SL252	2018	Human	Blood	SAMEA113605785	2018-01-707	Kp1	SL290	2018	Turkey	Cecal	SAMEA8399469
NK_H21_005	Kp1	SL11101	2017	Human	Blood	SAMEA113605794	2018-01-778	Kp1	SL17	2018	Turkey	Cecal	SAMEA8399476
NK_H21_010	Kp1	SL35	2017	Human	Blood	SAMEA113605798	2018-01-880	Kp1	SL432	2018	Turkey	Cecal	SAMEA8399486
NK_H21_011	Kp1	SL6	2017	Human	Blood	SAMEA113605799	KGVET-2020-01-126	Kp1	SL1628	2020	Turkey	Cecal	SAMN32676047
NK_H21_014	Kp1	SL359	2017	Human	Blood	SAMEA113605802	KGVET-2020-01-202-1	Kp1	SL2097	2020	Turkey	Cecal	SAMN32676060
NK_H21_015	Kp1	SL107	2017	Human	Blood	SAMEA113605031	KGVET-2020-01-2067-1	Kp1	SL49	2020	Turkey	Cecal	SAMN32676061
NK_H21_023	Kp1	SL25	2017	Human	Blood	SAMEA113605810	KGVET-2020-01-2158-1	Kp1	SL290	2020	Turkey	Cecal	SAMN32676062
NK_H21_029	Kp1	SL873	2017	Human	Blood	SAMEA113605816	KGVET-2020-01-2209-1	Kp1	SL290	2020	Turkey	Cecal	SAMN32676063
NK_H21_036	Kp1	SL107	2017	Human	Blood	SAMEA113605823	KGVET-2020-01-236-2	Kp1	SL37	2020	Turkey	Cecal	SAMN32676069
NK_H21_037	Kp1	SL307	2017	Human	Blood	SAMEA113605824	KGVET-2020-01-2496-3	Kp1	SL10561	2020	Turkey	Cecal	SAMN32676075
NK_H21_049	Kp4	SL367	2018	Human	Blood	SAMEA113605832	KGVET-2020-01-2661	Kp1	SL152	2020	Turkey	Cecal	SAMN32676082
NK_H21_050	Kp3	SL10052	2018	Human	Blood	SAMEA113605833	KGVET-2020-01-2677-1	Kp1	SL10178	2020	Turkey	Cecal	SAMN32676084
NK_H21_056A	Kp3	SL3201	2018	Human	Blood	SAMEA113605370	KGVET-2020-01-2800-1	Kp1	SL1999	2020	Turkey	Cecal	SAMN32676085
NK_H21_067	Kp3	SL11075	2018	Human	Blood	SAMEA113605847	KGVET-2020-01-2959-1	Kp1	SL13	2020	Turkey	Cecal	SAMN32676095
NK_H21_076	Kp1	SL301	2018	Human	Blood	SAMEA113605855	KGVET-2020-01-3043-2	Kp1	SL17	2020	Turkey	Cecal	SAMN32676100
NK_H21_078	Kp1	SL17	2018	Human	Blood	SAMEA113605857	KGVET-2020-01-3421-1	Kp1	SL10873	2020	Turkey	Cecal	SAMN32676111
NK_H21_080	Kp3	SL10826	2018	Human	Blood	SAMEA113605346	KGVET-2020-01-3721-3	Kp1	SL1628	2020	Turkey	Cecal	SAMN32676123
NK_H22_009	Kp3	SL11093	2016	Human	Blood	SAMEA114492469	KGVET-2020-01-3775-3	Kp1	SL10114	2020	Turkey	Cecal	SAMN32676124
NK_H22_013	Kp1	SL17	2017	Human	Blood	SAMEA114492473	KGVET-2020-01-4249-1	Kp1	SL10538	2020	Turkey	Cecal	SAMN32676138
NK_H22_015	Kp1	SL11306	2017	Human	Blood	SAMEA114492475	KGVET-2020-04-18146	Kp1	SL10475	2020	Turkey	Gizzard	SAMEA115163000

^*a*^ Kp1, *Klebsiella pneumoniae*; Kp2, *K. quasipneumoniae* subsp. *quasipneumoniae*; Kp3, *K. variicola*; Kp4, *K. quasipneumoniae* subsp. *similipneumoniae*; Kp6, *K. quasivariicola*.

## Data Availability

The genomes have been deposited under the umbrella BioProject no. PRJEB74192. Sequencing details, metadata, and accession numbers for the hybrid assemblies and Illumina and ONT reads are listed in Table 1. The full version of Table 1 is available on Figshare at https://doi.org/10.6084/m9.figshare.c.7622345.

## References

[1] Thorpe HA, Booton R, Kallonen T, Gibbon MJ, Couto N, Passet V, López-Fernández S, Rodrigues C, Matthews L, Mitchell S, Reeve R, David S, Merla C, Corbella M, Ferrari C, Comandatore F, Marone P, Brisse S, Sassera D, Corander J, Feil EJ. 2022. A large-scale genomic snapshot of Klebsiella spp. isolates in Northern Italy reveals limited transmission between clinical and non-clinical settings. Nat Microbiol 7:2054–2067. doi:10.1038/s41564-022-01263-0 36411354 PMC9712112

[2] Fostervold A, Hetland MAK, Bakksjø R, Bernhoff E, Holt KE, Samuelsen Ø, Simonsen GS, Sundsfjord A, Wyres KL, Löhr IH, et al.. 2022. A nationwide genomic study of clinical Klebsiella pneumoniae in Norway 2001–15: introduction and spread of ESBLs facilitated by clonal groups CG15 and CG307 . J Antimicrob Chemother 77:665–674. doi:10.1093/jac/dkab463 34935048 PMC8865009

[3] Fostervold A, Raffelsberger N, Hetland MAK, Bakksjø R, Bernhoff E, Samuelsen Ø, Sundsfjord A, Afset JE, Berntsen CF, Bævre-Jensen R, Ebbesen MH, Gammelsrud KW, Guleng AD, Handal N, Jakovljev A, Johal SK, Marvik Å, Natvik A, Sandnes R-A, Tofteland S, Bjørnholt JV, Löhr IH, Assoc. on behalf of The Norwegian Study Group on *Klebsiella pneumoniae*. 2024. Risk of death in Klebsiella pneumoniae bloodstream infections is associated with specific phylogenetic lineages. J Infect 88:106155. doi:10.1016/j.jinf.2024.106155 38574775

[4] Raffelsberger N, Hetland MAK, Svendsen K, Småbrekke L, Löhr IH, Andreassen LLE, Brisse S, Holt KE, Sundsfjord A, Samuelsen Ø, Gravningen K. 2021. Gastrointestinal carriage of Klebsiella pneumoniae in a general adult population: a cross-sectional study of risk factors and bacterial genomic diversity. Gut Microbes 13:1939599. doi:10.1080/19490976.2021.1939599 34182896 PMC8244762

[5] Franklin-Alming FV, Kaspersen H, Hetland MAK, Bakksjø RJ, Nesse LL, Leangapichart T, Löhr IH, Telke AA, Sunde M. 2021. Exploring Klebsiella pneumoniae in healthy poultry reveals high genetic diversity, good biofilm-forming abilities and higher prevalence in turkeys than broilers. Front Microbiol 12:725414. doi:10.3389/fmicb.2021.725414 34557173 PMC8453068

[6] Kaspersen H, Urdahl AM, Franklin-Alming FV, Ilag HK, Hetland MAK, Bernhoff E, Löhr IH, Sunde M. 2023. Population dynamics and characteristics of Klebsiella pneumoniae from healthy poultry in Norway. Front Microbiol 14:1193274. doi:10.3389/fmicb.2023.1193274 37275151 PMC10232788

[7] Kaspersen H, Franklin-Alming FV, Hetland MAK, Bernhoff E, Löhr IH, Jiwakanon J, Urdahl AM, Leangapichart T, Sunde M. 2023. Highly conserved composite transposon harbouring aerobactin iuc3 in Klebsiella pneumoniae from pigs. Microb Genom 9. doi:10.1099/mgen.0.000960 PMC9997749 36820818

[8] Håkonsholm F, Hetland MAK, Svanevik CS, Sundsfjord A, Lunestad BT, Marathe NP. 2020. Antibiotic sensitivity screening of Klebsiella spp. and Raoultella spp. isolated from marine bivalve molluscs reveal presence of CTX-M-Producing K. pneumoniae. Microorganisms 8:1909. doi:10.3390/microorganisms8121909 33266320 PMC7761178

[9] Håkonsholm F, Hetland MAK, Svanevik CS, Lunestad BT, Löhr IH, Marathe NP. 2022. Insights into the genetic diversity, antibiotic resistance and pathogenic potential of Klebsiella pneumoniae from the Norwegian marine environment using whole-genome analysis. Int J Hyg Environ Health 242:113967. doi:10.1016/j.ijheh.2022.113967 35398801

[10] Håkonsholm F, Hetland MAK, Löhr IH, Lunestad BT, Marathe NP. 2023. Co-localization of clinically relevant antibiotic- and heavy metal resistance genes on plasmids in Klebsiella pneumoniae from marine bivalves. Microbiologyopen 12:e1368. doi:10.1002/mbo3.1368 37642489 PMC10356976

[11] Kolmogorov M, Yuan J, Lin Y, Pevzner PA. 2019. Assembly of long, error-prone reads using repeat graphs. Nat Biotechnol 37:540–546. doi:10.1038/s41587-019-0072-8 30936562

[12] Wick RR, Judd LM, Gorrie CL, Holt KE. 2017. Unicycler: resolving bacterial genome assemblies from short and long sequencing reads. PLoS Comput Biol 13:e1005595. doi:10.1371/journal.pcbi.1005595 28594827 PMC5481147

[13] Johnson J, Soehnlen M, Blankenship HM. 2023. Long read genome assemblers struggle with small plasmids. Microb Genom 9:001024. doi:10.1099/mgen.0.001024 PMC10272865 37224062

[14] Wick RR, Holt KE. 2022. Polypolish: short-read polishing of long-read bacterial genome assemblies. PLOS Comput Biol 18:e1009802. doi:10.1371/journal.pcbi.1009802 35073327 PMC8812927

[15] Zimin AV, Salzberg SL. 2020. The genome polishing tool POLCA makes fast and accurate corrections in genome assemblies. PLoS Comput Biol 16:e1007981. doi:10.1371/journal.pcbi.1007981 32589667 PMC7347232

[16] Bankevich A, Nurk S, Antipov D, Gurevich AA, Dvorkin M, Kulikov AS, Lesin VM, Nikolenko SI, Pham S, Prjibelski AD, Pyshkin AV, Sirotkin AV, Vyahhi N, Tesler G, Alekseyev MA, Pevzner PA. 2012. SPAdes: a new genome assembly algorithm and its applications to single-cell sequencing. J Comput Biol 19:455–477. doi:10.1089/cmb.2012.0021 22506599 PMC3342519

[17] Wick RR, Judd LM, Cerdeira LT, Hawkey J, Méric G, Vezina B, Wyres KL, Holt KE. 2021. Trycycler: consensus long-read assemblies for bacterial genomes. Genome Biol 22:266. doi:10.1186/s13059-021-02483-z 34521459 PMC8442456

[18] Hunt M, Silva ND, Otto TD, Parkhill J, Keane JA, Harris SR. 2015. Circlator: automated circularization of genome assemblies using long sequencing reads. Genome Biol 16:294. doi:10.1186/s13059-015-0849-0 26714481 PMC4699355

[19] Gurevich A, Saveliev V, Vyahhi N, Tesler G. 2013. QUAST: quality assessment tool for genome assemblies. Bioinformatics 29:1072–1075. doi:10.1093/bioinformatics/btt086 23422339 PMC3624806

[20] Lam MMC, Wick RR, Watts SC, Cerdeira LT, Wyres KL, Holt KE. 2021. A genomic surveillance framework and genotyping tool for Klebsiella pneumoniae and its related species complex. Nat Commun 12:4188. doi:10.1038/s41467-021-24448-3 34234121 PMC8263825

[21] Tatusova T, DiCuccio M, Badretdin A, Chetvernin V, Nawrocki EP, Zaslavsky L, Lomsadze A, Pruitt KD, Borodovsky M, Ostell J. 2016. NCBI prokaryotic genome annotation pipeline. Nucleic Acids Res 44:6614–6624. doi:10.1093/nar/gkw569 27342282 PMC5001611

[22] Tonkin-Hill G, MacAlasdair N, Ruis C, Weimann A, Horesh G, Lees JA, Gladstone RA, Lo S, Beaudoin C, Floto RA, Frost SDW, Corander J, Bentley SD, Parkhill J. 2020. Producing polished prokaryotic pangenomes with the Panaroo pipeline. Genome Biol 21:180. doi:10.1186/s13059-020-02090-4 32698896 PMC7376924

